# New Method of Microimages Generation for 3D Display

**DOI:** 10.3390/s18092805

**Published:** 2018-08-25

**Authors:** Nicolò Incardona, Seokmin Hong, Manuel Martínez-Corral, Genaro Saavedra

**Affiliations:** Department of Optics, University of Valencia, 46100 Burjassot, Valencia, Spain; seokmin.hong@uv.es (S.H.); manuel.martinez@uv.es (M.M.-C.); genaro.saavedra@uv.es (G.S.)

**Keywords:** integral imaging, plenoptic imaging, 3D display, CGII

## Abstract

In this paper, we propose a new method for the generation of microimages, which processes real 3D scenes captured with any method that permits the extraction of its depth information. The depth map of the scene, together with its color information, is used to create a point cloud. A set of elemental images of this point cloud is captured synthetically and from it the microimages are computed. The main feature of this method is that the reference plane of displayed images can be set at will, while the empty pixels are avoided. Another advantage of the method is that the center point of displayed images and also their scale and field of view can be set. To show the final results, a 3D InI display prototype is implemented through a tablet and a microlens array. We demonstrate that this new technique overcomes the drawbacks of previous similar ones and provides more flexibility setting the characteristics of the final image.

## 1. Introduction

**Figure 1:**
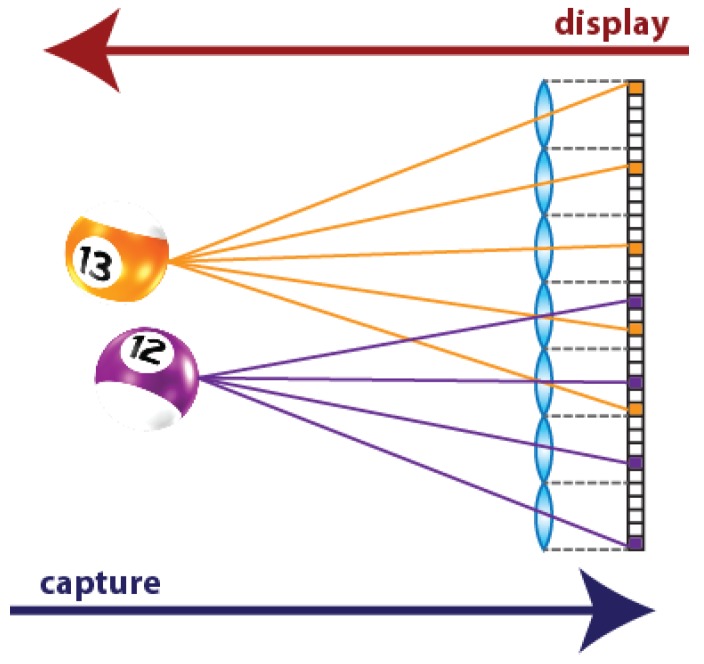
A 3D scene captured and displayed with IP technique. Note that, in the capturing stage, the observer is in the right side (behind the camera’s sensor), so he sees the violet ball closer. In the display stage, the observer is in the left side (in front of the MLA of the InI display), so he sees the orange ball closer: the scene is reconstructed with reversed depth.

3D TV implementation is a fascinating challenge for the researchers of many different scientific communities. The first generation of 3D TV is no longer produced: one of the main drawbacks of these devices was the need of glasses in order to see the 3D content. For this reason, a new type of glasses-free device is being investigated, the so-called autostereoscopic displays. Among these, multi-view displays allow 3D visualization for multiple viewers with stereo and movement parallax, and overcome the accommodation-convergence conflict [1,2]. This kind of displays are named Integral Photography (IP) or Integral Imaging (InI) displays, because their operating principle is based on the Integral Photography technique. It was proposed one century ago by Gabriel Lippmann to register the 3D information of a scene [3]. His idea was to replace the objective lens of the photographic camera with a microlens array (MLA), and to place a photographic film at the focal plane of the lenses. Doing so, different perspectives of the scene are captured. The part of the photographic film (nowadays, the portion of the pixelated sensor) behind each microlens corresponds to a different perspective. These perspective views are called Elemental Images (EIs) and the set of EIs is the Integral Image (InI). The aim of Lippmann was to project the images captured with IP through a MLA similar to the one used in the capturing stage. Doing so, the light emitted by the EIs of the photographic film is integrated in front of the MLA, producing a 3D reconstruction of the original captured scene (Figure 1).

The great progress in optoelectronic technologies renewed the interest in this technique. Commercial cameras based on IP, known as plenoptic cameras, are already available on the market [4]. These cameras give access to a great number of applications such as the extraction of the depth map, or digital refocusing of the picture. Moreover, the continuous advances in displays (4 K and even 8 K displays are already available on the market) and in MLA manufacturing are a great boost for research in 3D InI displays.

It is important to remark that the EIs captured through IP technique are not directly projectable in the 3D InI monitor. Firstly, because the MLA used in the capturing process is not the same of that used in the display. Moreover, if EIs are directly projected, the reconstructed scene will be a pseudoscopic version of the original one, which means that the scene is reconstructed with reversed depth, as shown in Figure 1. To solve these problems, some computations have to be made, to convert the EIs into the so-called microimages. The conversion is made by means of a simple transposition, that is, through a pixel resampling [5].

The proposed method generates and processes the EIs to convert them to microimages. It overcomes the drawbacks of previous ones and is applicable to real scenes. The operating principle is very simple: the 3D scene is firstly captured and converted to a virtual point cloud. Any method that permits the extraction of a 2D depth map of the scene can be used for this task. Then, the EIs are synthetically captured from this point cloud through a virtual cameras array. Finally, the EIs are processed and converted to microimages projectable in the 3D InI display. Since the EIs are synthetically captured, we have much more freedom in adjusting the parameters of the cameras array. In this way, it is possible to change the characteristics of the final image and the way the scene is reconstructed by the 3D InI display. Above all, it is possible to adapt the algorithm to any InI display, without having to repeat the capture of the scene. A geometrical model of the system is exploited, which permits directly setting with high precision the reference plane’s position and the field of view of the image. The reference plane’s position is fundamental because it sets the portion of the 3D scene that will be reconstructed inside and outside the InI monitor, changing the depth sensation of the scene. The part of the scene that is behind the reference plane is reconstructed by the MLA as a virtual image inside the screen, while the part that is beyond the reference plane is reconstructed as a real image, floating in front of the screen.

This paper is organized as follows. In Section 2, a quick review on the previous work on techniques for microimages generation is done. In Section 3, our new technique is described, and the geometrical model of the system is explained. In Section 4, the experimental results obtained are presented. Finally, in Section 5, the achievements of the presented work are summarized.

## 2. Previous Work

As stated before, the EIs captured with Lippmann’s technique are not directly projectable in the InI monitor. Many methods to generate microimages for InI displays have already been reported. Kwon et al. [6], Jiao et al. [7] and Li et al. [8] used different techniques to generate synthetically the EIs, and then process them to obtain the microimages. Instead, Chen et al. [9] directly captured the microimages putting a virtual pinhole array (VPA) into the 3D model. However, all these methods only process computer-generated 3D models, not real world 3D scenes.

Among the techniques that process real 3D scenes, Navarro et al. [10] and Martínez-Corral et al. [11] proposed the so-called Smart Pseudoscopic-to-Orthoscopic Conversion (SPOC). A collection of EIs is optically captured. Then, through a smart pixel mapping, the microimages are obtained, with the possibility to change the reference plane’s position. This method has some limitations. First, if the synthetic aperture method is used to capture the EIs (EIs captured with a single conventional camera mechanically displaced), the capturing stage is very time-consuming. Moreover, the reference plane’s position can be set only at determined planes, because just one parameter can be used for this task.

Hong et al. (2015) [12] and Hong et al. (2018) [13] used Kinect cameras to capture the spatial and depth information of the scene. Then, these data are merged into a point cloud. A VPA is used to directly obtain the microimages adjusted to the InI monitor. With this method, the capturing stage is reduced to a single snapshot of the scene. Besides, the amount of data to process is greatly reduced: an RGB image and a depth map are sufficient. However, another issue appears. The VPA used to generate the microimages is set near or directly inside the scene. The point cloud has a finite number of elements: the ones that are close to the VPA have a very big angle with respect to the pinholes, so they do not map onto any pixel. For this reason, this part of information of the scene is lost and large areas with black pixels, that is pixels with no information, appear in the final image.

Piao et al. [14] and Cho and Shin [15] used off-axially distributed image sensing (ODIS) and axially distributed image sensing (ADS), respectively, to extract the color and depth information of the 3D scene. Again, this information is used to compute synthetically the microimages through a VPA.

The proposed technique overcomes black pixels’ drawback and it offers much more flexibility than the mentioned ones.

## 3. Proposed Technique

The basic idea is to capture the real 3D scene and convert it to a virtual point cloud in order to process it synthetically. A set of EIs of the virtual 3D scene (the point cloud) is captured through a simulated cameras array, and processed as in [11] to obtain the microimages. The EIs are synthetically captured from a virtual point cloud and not optically captured from the real scene. Doing so, without repeating the real scene capturing step, one can change the characteristics of the integral image: the number of horizontal and vertical EIs, the amount of parallax and the field of view. All can be set modifying the simulated cameras array.

### 3.1. Microimages Generation Process

**Figure 2:**
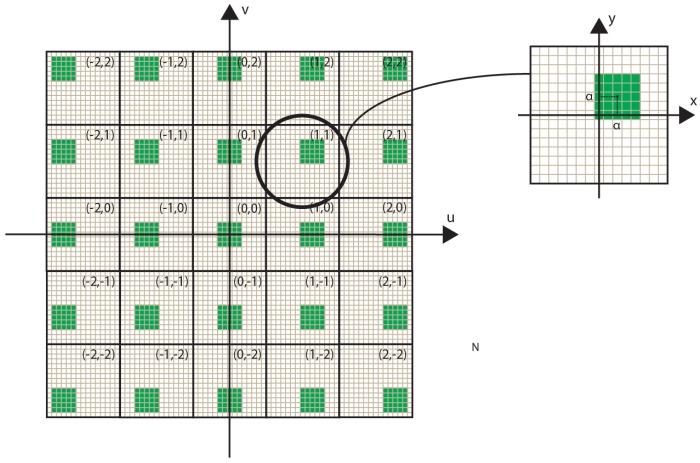
The EIs cropping. In the classical (*u*, *v*, *x*, *y*) parameterization of the integral image, let us say the central EI’s coordinates are (*u_0_*, *v_0_*) and the central pixel’s coordinates of every EI are (*x_0_*, *y_0_*). Considering the EI having coordinates (*u*, *v*), the central pixel of its sub-EI has coordinates (*x*, *y*) = (*x_0_* + (*u* −*u_0_*)α, *y_0_* + (*v*−*v_0_*)α).

**Figure 3:**
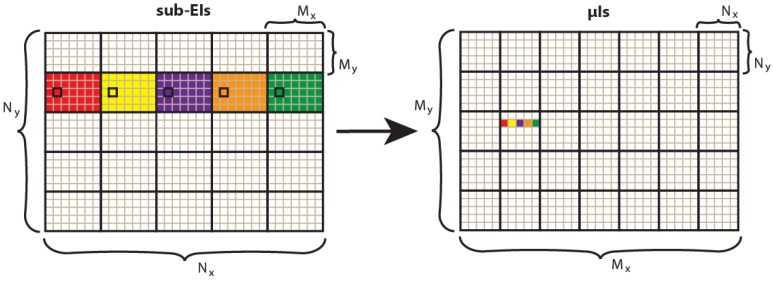
Transposition from sub-EIs to microimages. Starting from (*N_x_* ×*N_y_*) sub-EIs, each one with (*M_x_*×*M_y_*) pixels, we obtain (*M_x_*×*M_y_*) microimages each one with (*N_x_*×*N_y_*) pixels. The correspondence is: *p_i,j_(μI_k,l_)* = *p_k,l_(sub-EI_i,j_)*, where *p* means pixel.

The process can be divided into five steps:

1. *Point Cloud creation.* The scene is captured and its depth information is extracted. A point cloud representing the scene is generated merging the RGB and depth information.
2. *EIs capturing.* The EIs are generated using a VPA. The number of virtual pinhole cameras in vertical (horizontal) direction is set equal to the number of pixels behind each microlens of the InI monitor in vertical (horizontal) direction. To capture EIs, the VPA is placed far away from the scene. A trade-off between the resolution of the EIs and the absence of black pixels depends on the position of the VPA. If the VPA is set too close to the point cloud, some information of the scene is lost and black pixels appear in the EIs (for the same reason as in [12,13]). On the other hand, as the VPA is moved further from the point cloud, black pixels’ issue disappears, but the scene is captured in the EIs with lower resolution. Therefore, the position of the VPA is set empirically at the minimum distance from the point cloud that ensures the absence of black pixels. Then, this value is refined to set the reference plane’s position, as explained in Section 3.2.
3. *Shifted cropping.* A portion of (*L* × *V*) pixels of every EIs is cropped as in Figure 2. Shifting the cropped region with a constant step between adjacent EIs, allows setting the reference plane of the final image. This sets the portion of the 3D scene that will be reconstructed inside and outside the InI display, changing the depth sensation. More details on the parameters used in this step are given in Section 3.2.
4. *Resize.* The cropped EIs (sub-EIs) are resized to the spatial resolution of the InI monitor, that is, the number of microlenses of the MLA in horizontal and vertical direction.
5. *Transposition.* The pixels are resampled as in Figure 3, to convert the sub-EIs to the final microimages to project in the 3D InI monitor.

### 3.2. Geometrical Model

One great feature of this algorithm is that, exploiting a geometrical model of the system, its parameters are adjusted to precisely set the position of the reference plane and the field of view of the final image.

**Figure 4:**
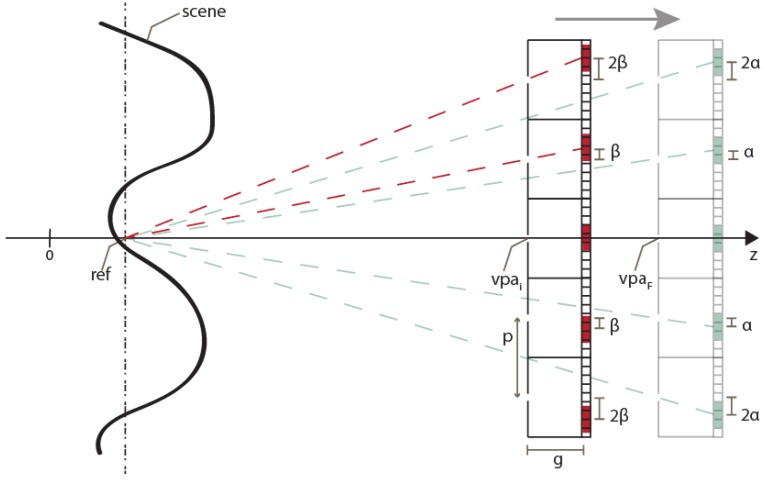
The procedure to set the reference plane’s position. Note that, for graphical convenience, a VPA with pitch equal to the dimension of a single pinhole camera is shown. Actually, the pitch is smaller, so the virtual pinhole cameras overlap with each other.

To set the reference plane’s position, the distance of the VPA and the shifting factor of the sub-EIs (α in Figure 2) can be adjusted. The procedure is the following. First, the VPA’s position is set to the initial value *vpa_i_*. As explained above, it has to be far enough from the scene to avoid black pixels’ issue. Then, a provisional value of the shifting factor of the sub-EIs is calculated (β in Figure 4). As shown in Figure 4, it is the number of pixels of shifting which makes all the sub-EIs converge on the plane at *z* = *ref*

$$\beta =\frac{p\times g}{{vpa}_{i}-ref}$$

where *p* is the pitch between the cameras and *g* is the gap of the VPA. As the number of pixels of shifting must obviously be an integer number, the value of β must be rounded:

α=round(β)

Now, if we consider these values of *vpa* and α, the reference plane would not be at *z* = *ref*. Therefore, we have to move the VPA’s position to

$${vpa}_{f}=\frac{p\times g}{\alpha }+ref$$

Operating on these two parameters (α and *vpa*), the reference plane’s position can be set with very high resolution and precision. In [11], only α could be changed, because the distance of the cameras is fixed in the optical capturing stage. Thus, the image can be reconstructed only at determined planes, which depend on the physical and optical parameters of the real scene capture.

**Figure 5:**
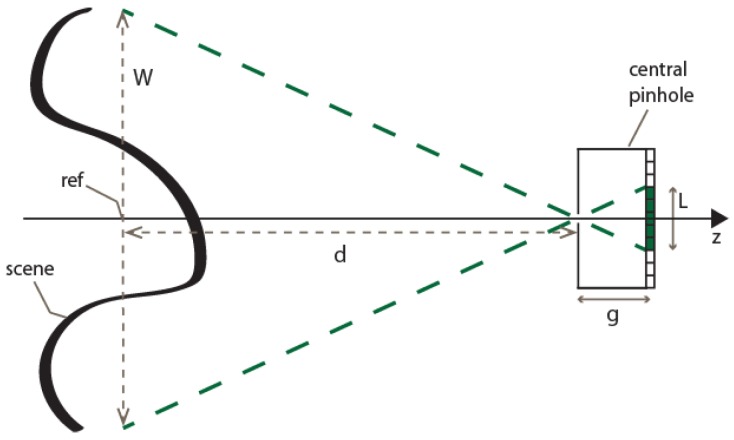
Setting the cropping factor. To set the number of pixels of the sub-EIs, a simple geometrical relation can be exploited: $L=\frac{g\times W}{d}$ (obviously, *L* has to be rounded to the nearest integer number). Reducing *L*, we can reduce the field of view.

The parameter that sets the field of view of the final image is the number of pixels of the sub-EIs. Let us assume, for instance, that we want to represent all the useful scene given by a point cloud having a width *W*. As we can see in Figure 5, the width of the cropped area has to be

$$L=\frac{g\times W}{d}$$

with *d* being the distance between the VPA and the reference plane. If we want to reduce the field of view to a 1/*z* portion of the scene, it is sufficient to divide by *z* the previous expression of *L*. The number of pixels in the other dimension (in vertical direction, if we assume that *L* is the number of pixels in horizontal direction) depends on the resolution of the InI display:

$$V=\frac{R_{y}}{R_{x}}L$$

with *R_y_* and *R_x_* being the vertical and horizontal spatial resolution of the InI display respectively. As explained in the previous section, the sub-EIs are finally resized from (*L* ×*V*) to (*R_x_*× *R_y_*), before the final transposition.

## 4. Experimental Results

**Figure 6:**
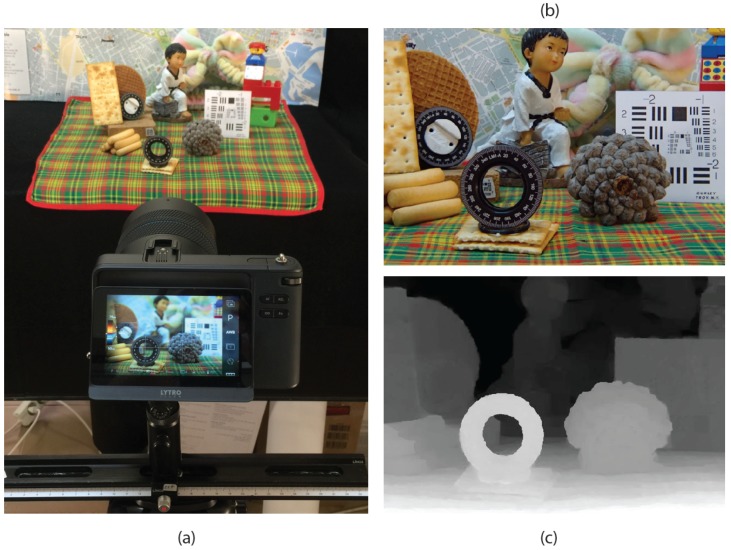
(**a**) Experimental set-up of the real capture of the 3D scene. (**b**) The RGB image extracted from Lytro Desktop software. (**c**) The depth map of the scene extracted from Lytro Desktop software.

To demonstrate the effectiveness of the proposed method, we applied it to a real 3D scene that we captured with a Lytro Illum plenoptic camera. The experimental setup is shown in Figure 6a. The RGB image and the depth map (Figure 6b,c) are extracted through the Lytro Desktop software.

For the implementation of the 3D InI monitor, we used a Samsung SM-T700 tablet, with a screen of 14.1492 pixels/mm (ppm), and a MLA (Fresneltech, model 630) composed by lenslets of focal length *f* = 3.3 mm and pitch *p* = 1.0 mm. We have exactly 14.1492 pixels per microlens, thus, in the algorithm, we set the VPA to have 15 × 15 virtual pinhole cameras. In the resize stage, the sub-EIs are resized to 151 × 113 to make maximum use of the MLA in horizontal direction (the MLA is square-shaped, with a side of 151 mm, so it has 151 × 151 lenslets). Doing so, the final image will be 2265 × 1695. Nevertheless, it is important to remark the fact that the real number of pixels per microlens is 14.1492, so the image is finally rescaled to 2136 × 1599 (rescale factor *k* = 14.1492/15).

**Figure 7:**
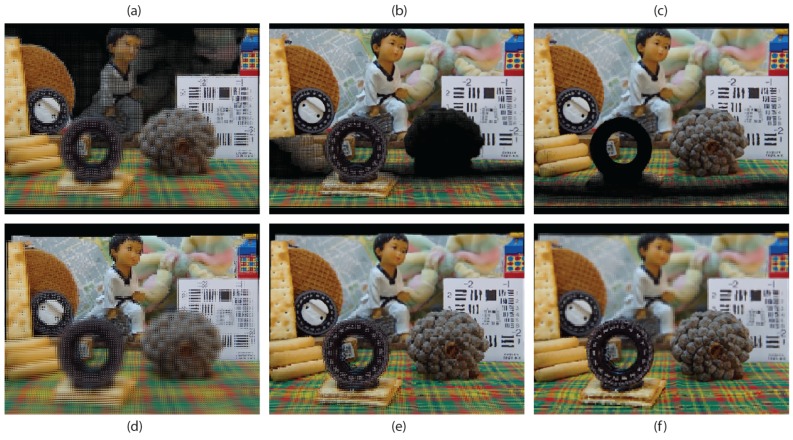
Top row: Images obtained with the technique of [12]. Bottom row: Images obtained with the proposed technique. (**a**,**d**) Reference plane at the background; (**b**,**e**) reference plane at the middle; and (**c**,**f**) reference plane at the foreground.

**Figure 8:**
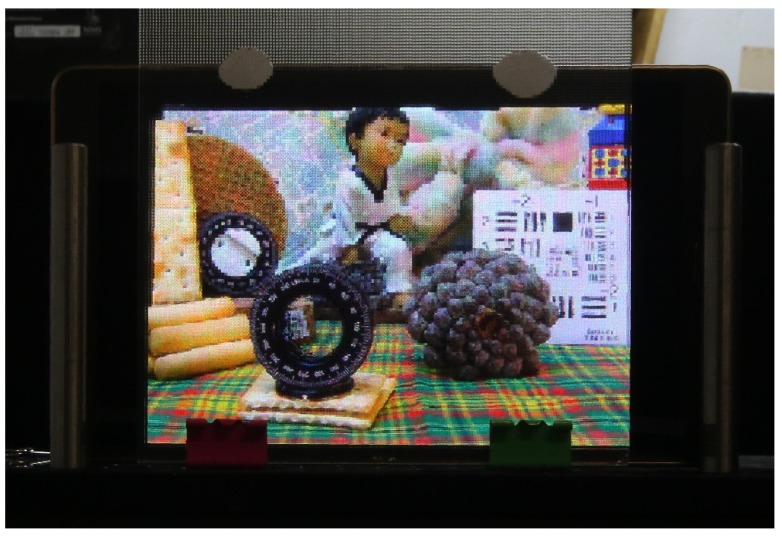
The integral image of Figure 7d projected in our 3D InI display prototype.

Figure 7 presents a comparison between the results obtained with the proposed technique and with the technique of [12], using the same point cloud as input. In the top row, the three images generated with the latter, with the reference plane set at three different depths are shown. In the bottom row, the images obtained with the proposed technique, with the reference plane set exactly at the same depths of the corresponding images of the top row. Clearly, in the images obtained with the concurrent method, a large black area (no information area) appears in the region close to the reference plane’s position. Instead, in the images obtained with the proposed method, this problem does not occur. There are just some black pixels due to occlusions or bad depth estimation. Figure 8 shows the image of Figure 7d projected in our 3D InI display prototype. The reference plane is set at the background, so the map, the doll and the colored bow are reconstructed on the MLA plane, while the rest of the scene is reconstructed floating in front of it. Video S1 (https://youtu.be/X9UJGEh4CdE) is a video recording the InI display, which is much more effective than a single picture to perceive the 3D sensation of the reconstructed scene.

**Figure 9:**
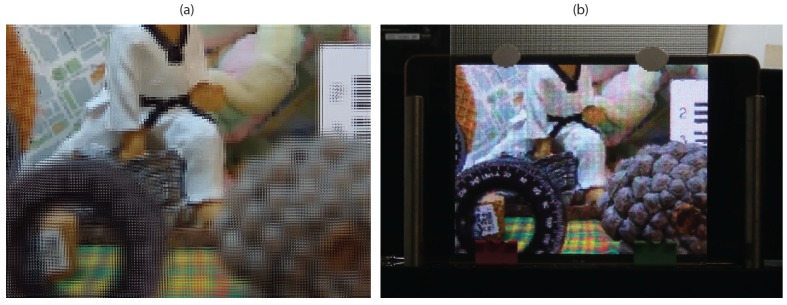
Field of view selection: (**a**) The integral image obtained with the same reference plane and pitch of Figure 7d, with half its field of view (*z* = 2). (**b**) Projection in 3D InI display.

In Figure 9, the field of view is reduced by a factor *z* = 2. The image obtained is shown in Figure 9a, while in Figure 9b we show the image projected in the InI display.

**Figure 10:**
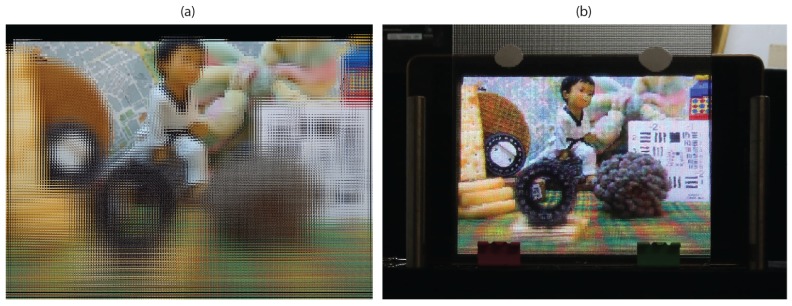
Pitch selection: (**a**) The integral image obtained with the same reference plane and field of view of Figure 7d, with five times its pitch. The DOF is greatly reduced with respect to the image of Figure 7d. (**b**) Projection in 3D InI display. The objects appear increasingly defocused as we move far from the reference plane.

In Figure 10, the pitch *p* of the pinhole cameras of the VPA is increased by a factor 5. The reference plane is set at the same position of the image of Figure 7d. Increasing the pitch has a double effect: the parallax amount increases while the depth of field (DOF) of the image decreases. On the contrary, setting an excessively low value of *p* increases the DOF, but the scene is reconstructed very flatly and with low parallax, thus losing the 3D sensation. The DOF of the image has to be adjusted to the DOF of the InI monitor to obtain a good reconstruction of the 3D scene. Here, *p* is intentionally set to a very high value in order to show the effect of an excessively large pitch. In Figure 10a, the integral image is shown. Comparing with Figure 7d, the DOF is greatly reduced: only the background map is in focus, while the rest of the objects appear increasingly defocused as we move away from the reference plane. In Figure 10b, the image projected in the InI monitor is shown. Note that the objects that are close to the reference plane are reconstructed well by the InI monitor, while the ones that are far from it appear really defocused. For matter of comparison, in Figure 8, which shows the image obtained with a fair value of *p* projected in the InI monitor, the whole scene is reconstructed well.

## 5. Conclusions and Future Work

We have proposed a new method for the generation of microimages projectable in 3D InI displays. The method works with real 3D scenes and is adaptable to any InI display. It solves the problem of black pixels’ areas and allows setting with precision the field of view of the final image and its reference plane, so controlling the way the scene is reconstructed by the InI monitor.

In the future work, the main focus will be on the real-time implementation of the system. Capturing the EIs through the VPA is the most time-consuming step, so it must be reassessed. Another goal is to resolve occlusions by using the information of multiple views of the integral image of the Lytro. The idea is to extract the depth maps of the lateral EIs and merge the RGB with the depth information of all these views into a single point cloud.
